# Validity and Reliability of the WIMU^TM^ Inertial Device for the Assessment of Joint Angulations

**DOI:** 10.3390/ijerph17010193

**Published:** 2019-12-27

**Authors:** Javier García-Rubio, José Pino, Pedro R. Olivares, Sergio J. Ibáñez

**Affiliations:** 1 Faculty of Sports Science, University of Extremadura, Optimization of Training and Sport Performance Research Group, 10005 Cáceres, Spain; sibanez@unex.es; 2 Facultad de Educación, Universidad Autónoma de Chile, Santiago de Chile 7500000, Chile; olivares.pedro@gmail.com; 3 Faculty of Sports Sciences, University of Murcia, 30001 San Javier, Spain; pepepinoortega@gmail.com; 4 Faculty of Education, University of Huelva, 21007 Huelva, Spain

**Keywords:** inertial device, angle assessment, dorsiflexion

## Abstract

Range of motion measurement is fundamental in the physical examination and functional evaluation of different joints. WIMU^TM^ is an inertial device that allows the analysis of joint motion easily in real time. This study had a two-fold goal: (i) to evaluate the validity of WIMU^TM^ on the measurement of different angle positions, compared with a standard goniometer and 2D video-based motion analysis software; and (ii) to evaluate the use of WIMU^TM^ in the assessment of angulations in a joint, specifically assessing the validity and reliability of WIMU^TM^ on the measurement of ankle dorsiflexion, compared to a standard goniometer and Kinovea. The intraclass correlation coefficient and Pearson´s correlation coefficient (r) were performed to calculate the concurrent validity, and Bland-Altman plots were performed to analyze agreement between measures. For the analysis of reliability, both relative and absolute indices were used. The results showed excellent validity and reliability of WIMU^TM^ in the assessment of angle positions and ankle dorsiflexion. The current findings conclude that WIMU^TM^ is a valid and reliable instrument to measure angle and joint motions. In short, WIMU^TM^ provides a new clinical and sportive method of angle measurement.

## 1. Introduction

An accurate and reliable measurement of the range of motion (ROM) is fundamental in the physical examination and functional evaluation of different joints, as well as in the monitoring and evaluation of training or rehabilitation programs. There are several instruments that have been used in the examination of ROM in different joints, such as visual observation, goniometers, linear measures, inclinometers [1], or even smartphones [2]. The gold standard in joint analysis is the three-dimensional (3D) motion analysis, but these instruments are expensive in terms of both time and money, thus not easily available to all professionals [3]. Two-dimensional (2D) motion analysis is commonly used due to its lower cost. Universal goniometers are the most used in clinical practice because its low cost, accessibility, ease of use, and portability [2]. On the contrary, a major drawback of goniometers is that they require the use of both of the user’s hands, increasing the risk of error in measurement, and have to be used in static positions [1]. Recently, technological advances in smartphone applications have increased the availability of a wide number of applications to measure ROM [2]. Goniometry-based apps (2D) use different mechanisms to calculate joint angles—DrGoniometer [4], Coach´s Eye [3], or Knee Goniometer App (KGA) [2]—which are reliable for use in clinical and athletic activities. 

In recent years, wireless inertial measurement units have been used as motion capture devices. These devices are based on inertial/magnetic systems that are used as a wearable device, allowing motion measurement. These devices data could be sent to a computer in real time to evaluate movement and give immediate feedback [5]. The use of wireless inertial measurement devices is increasing in research. However, their use in clinical practice is scarce, due to their price, compared with other instruments, and difficulty in analysis and interpretation of data by professionals not familiar with the specific software. Additionally, the latest global survey of fitness [6] stated that wearable technology will be the number one trend over the next few years, reinforcing the findings of previous surveys. Technological advances are unpredictable. For this reason, the validity and reliability of this technology should adapt to the expectations of new users, identifying difficulties in use and interpretation of reported data. 

Ankle dorsiflexion is commonly linked to several clinical conditions [7] and in sporting contexts [8]. In strength training exercises, such as squats, reduced ankle dorsiflexion decreases quadriceps activation and increases soleus initiation [9]. In daily activities, such as walking, descending stairs, (10°) [10], and running (20° to 30°) [11], a minimum ankle dorsiflexion is required. Based on its applicability in both clinical conditions and sporting contexts, a test for the assessment of ankle dorsiflexion was chosen to analyze the agreement between WIMU^TM^ measures and other commonly used devices (goniometer and 2D motion analysis). 

WIMU^TM^ is a wireless inertial device that was designed to control and monitor physical activity for performance, rehabilitation, and research. It can be used indoors or outdoors, with the advantage of being easy to handle, small, and lightweight. This inertial device consists of several sensors, such as accelerometers or gyroscopes, allowing the measurement of distance, speed and, specifically, angles. In addition, this device uses a specific software, QÜIKO^TM^, for automatic data analysis generated in real time. This software has different algorithms that automatically calculate varying measures and variables as angulations in a movement. In the QÜIKO menu, the user only has to choose the variable to analyze, and QÜIKO presents the results. WIMU^TM^ can be used in sporting contexts with dynamic movements, but in this work, because we compare goniometer and Kinovea results, only the static test was used.

Currently, instruments to measure joint ROM, specifically ankle dorsiflexion, was designed for static positions, allowing manually assessment (goniometer), or in a in a specific motion plane, not allowing the two-dimensional image (Kinovea and related software) to affect the measurements. Therefore, this study has a two-fold goal: (i) to evaluate the validity of WIMU^TM^ for the measurement of different angles positions, compared with two different methods; and (ii) to evaluate the validity and reliability of WIMU^TM^ for the measurement of ankle dorsiflexion, compared with the standard goniometer and Kinovea. We hypothesize that the WIMU^TM^ device will display high validity and reliability to measure different angulations, and the ankle dorsiflexion test used in the study.

## 2. Materials and Methods 

### 2.1. Instruments

WIMU^TM^ and two other measurement systems were used to assess angles: a standard goniometer and a video-based motion software analysis. 

WIMU^TM^ (RealTrack Systems, Almería, Spain): this device comprises of a variety of sensors (two tri-axial accelerometers (2G and 8G), 3D gyroscope, 3D magnetometer, barometer, and GPS). For the analysis of inclination angles, information from accelerometers, gyroscopes, and magnetometers is used to calculate unit quaternions, which are later transformed to Euler angles. All information was obtained and analyzed using its specific software, QÜIKO^TM^ (RealTrack Systems, Almería, Spain).

Gollehon Extendable Goniometer (model 01135, Lafayette Instrument Co., Lafayette, IN, USA): This goniometer has a dual scale of 0–180° and 180–0 with 1° increments, and has been used in previous studies to analyze the range of motion [12]. 

Kinovea (open source): This software is a free and open-source (GPL2) video player that allows motion analysis, including the calculation of angulations in real time, manually or using semi-automated tracking function. The reliability of this software in the analysis of motion range has been reported as high in both inter and intra-rater assessment. Kinovea can be downloaded from http://www.kinovea.org/.

### 2.2. Phase 1: Validity of WIMU^TM^ for the Measurement of Different Angle Positions

#### Procedure

**Figure 1:**
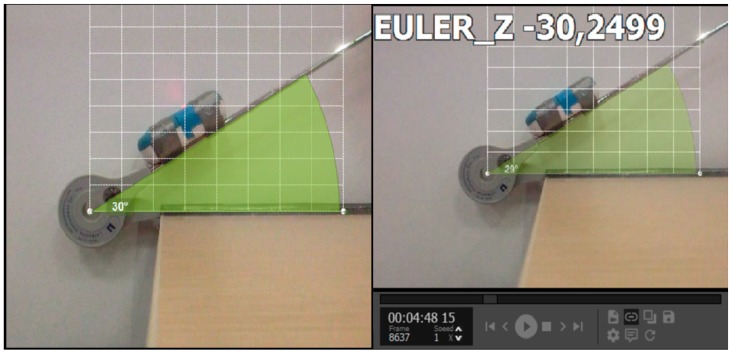
Procedure of Phase 1. The left image shows the inertial device attached to the goniometer, the position of the camera, and the automatic tracking function of the Kinovea calculating the angle. On the right, QÜIKO software automatically calculates the same angle.

The goniometer was fixed to a stable surface at 0°, while the other arm of the goniometer was kept free. The WIMU^TM^ was then attached to the free arm. The inertial device was automatically calibrated when switched on and simultaneously, a digital video camera (Sony EXILIM EX-ZR15, sampling frequency of 120 Hz) was aligned perpendicularly at the centre of the goniometer in a sagittal plane and at a distance of 30 cm (Figure 1). Both devices were synchronized, following which 88 aleatory angle measurements, in a range value of 0–180 degrees, were taken using the Lafayette Gollehon goniometer as reference.

The video images were imported to Kinovea and the semiautomatic tracking function allowed the calculation of angles in each position, providing information in real time. The information obtained by WIMU^TM^ was sent to the QÜIKOTM software, which automatically computed the angles. 

### 2.3. Phase 2: Validity and Reliability of WIMU^TM^ for the Measurement of Dorsiflexion Ankle

Participants: The participants in this study were 30 healthy male subjects (age = 17.0 ± 0.7; height = 1.7 ± 0.1; weight = 66.2 ± 8.4) from a U-18 level football club. The subjects and their parents were informed about the study procedures, and gave consent to participate. The participants were expected to have been free from lower extremity injury for six months prior to the testing, with no prior history of hip, knee, or ankle surgery. The ethics committee at the University of Extremadura approved the study (No. 67/2017).

**Figure 2:**
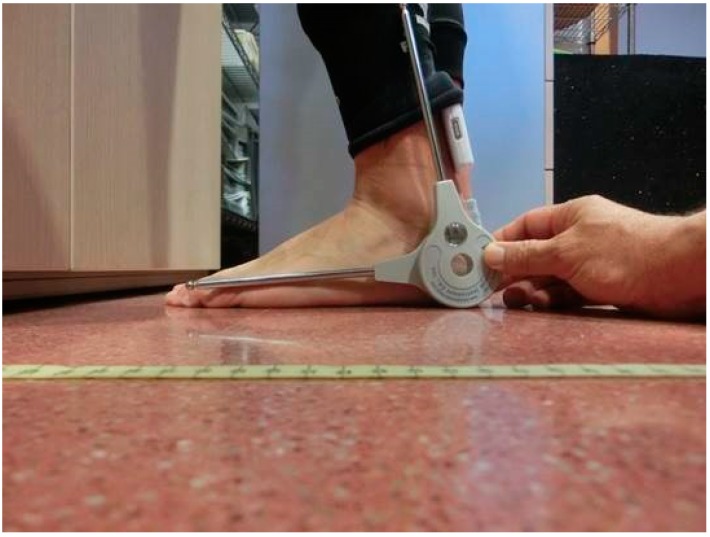
Measurement of ankle dorsiflexion with the knee in extension. WIMU^TM^ is attached to the Achilles tendon.

Procedure: The assessment of ankle dorsiflexion was conducted using a standardized protocol. Prior to the test, subjects perform a warm-up based on 8 to 10 min of jogging and static stretching, specifically of the lower-limbs [13]. All subjects were asked not to perform any intense physical activity 48 h before the test. Ankle dorsiflexion was recorded with the knee straight, both hands resting on a wall, and the heel kept in contact with the floor [7]. The WIMU^TM^ was placed on the calf muscle (Figure 2). The difference between the vertical angle (90°) and the measured angle was assessed as the dorsiflexion. The participants were asked to perform the test with one leg, doing three repetitions for each limb. Ankle dorsiflexion was assessed simultaneously using the WIMU^TM^, the goniometer, and Kinovea in order to check the agreement between these measures in this test. The standard goniometer was placed as literature suggests [8], aligned with the floor and visually bisecting the lateral malleolus and the fibular head. WIMU^TM^ was placed on the Achilles tendon, aligned with the tibia. 

In order to analyze the reliability of the data, the evaluation was conducted twice with the same participants, within a period of seven days.

### 2.4. Statistical Analysis

A descriptive analysis with mean and standard deviation was first carried out to characterize the measurements obtained for all devices in both phases. Data distribution was checked with the Kolmogorov-Smirnov test [14] to select the subsequent statistical analysis. Pearson’s correlation coefficient (r) was performed to calculate the strength of a relation between two variables [15]. Bland-Altman plots [14] were performed to complete the concurrent validity analysis of WIMU^TM^ with the representation of the degree of agreement between angles obtained using this device and both, goniometer and Kinovea software. The intraclass correlation coefficient (ICC) (2.1) was used for the analysis of the validity of angles measured between the WIMU^TM^ and Kinovea using the goniometer as reference. 

Relative reliability was estimated using the ICC (2.1) with 95% confidence intervals across the two test sessions [15]. ICC was interpreted according to Munro et al. as follows: moderate (0.50 to 0.69), high (0.70 to 0.89), and excellent (0.90 and above) [16]. Absolute reliability was determined by calculating the Standard Error of Measurement (SEM) (SEM = SD); where SD is the mean SD of test 1 and test 2), and the Smallest Real Difference (SRD) (1.96) [17].

The level of significance was established at *p* < 0.05. All of the analyses were performed using software SPSS 21.0 for Windows (IBM Co., Armonk, NY, USA), except for the Bland-Altman plots, which were made using the Graphpad Prism software (Graphpad, Inc., San Diego, CA, USA). 

## 3. Results

**Figure 3:**
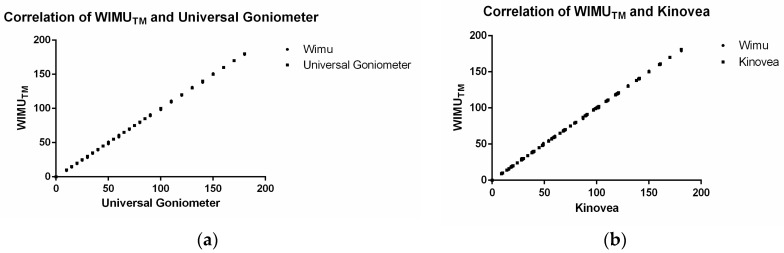
Agreement between Universal Goniometer (UG), Kinovea software, and WIMU^TM^ measurements, based on the measurement of different angles (*n* = 88) in Phase 1. (**a**) correlation of WIMU^TM^ and universal goniometer; (**b**) correlation of WIMU^TM^ and kinovea

**Figure 4:**
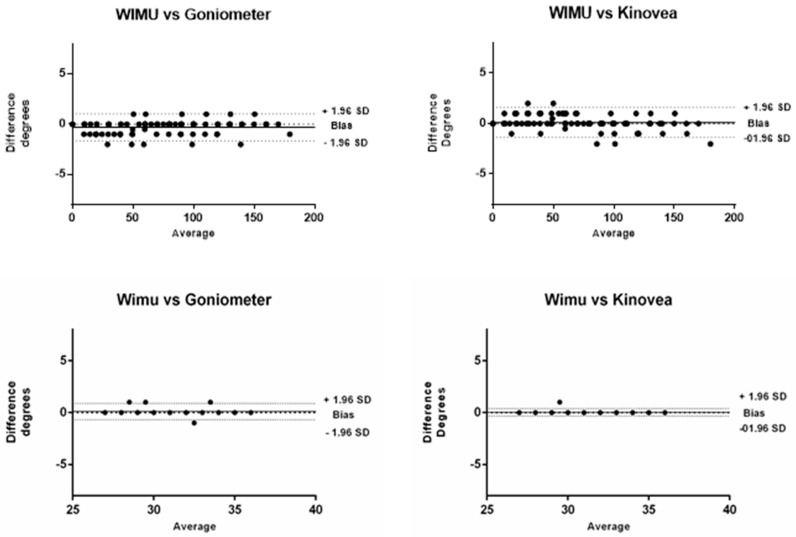
Bland-Altman plots with 95% limits of agreement between the WIMU^TM^ device, universal goniometer (**left panel**), and Kinovea (**right panel**) in Phase 1 (**upper**) and Phase 2 (**lower**).

Table 1 shows the means and standard deviations of the measures obtained by the WIMU^TM^ using Kinovea and universal goniometer as reference, as well as ICC and Pearson´s r values for the validity analysis for both Phase 1 and Phase 2. Values for convergent validity were excellent for both phases (>0.95), ICC and Pearson´s r indicating a perfect consistency between measures obtained by the three devices. Figure 3 showed the correlation between instruments in the first experiment. Bland-Altman plots (Figure 4) showed that most observations (>95%) were near the mean of the differences of instruments for both phases, 1.96 SD range of the differences. This analysis indicates that both instruments present a high degree of agreement. In addition, the plots show that in Phase 1, a trend of −0.30 and the limits of agreement (−1.63 and 1.02) are small enough for the validity of the measures, comparing WIMU^TM^ with the goniometer. When comparing WIMU^TM^ with Kinovea, a trend of 0.11 and limits of agreement (−1.36 and 1.59) show similar results of validity. This is the average difference between the two measures. Table 2 shows the relative and absolute reliability of the ankle dorsiflexion test for WIMU^TM^, Kinovea, and goniometer measurements. The ICC values were excellent for the three devices (all of them >0.90), and WIMU^TM^ showed the highest absolute reliability.

## 4. Discussion

The aim of this study was to evaluate the validity and reliability of WIMU^TM^ for the measurement of different angle positions, compared with a standard goniometer and a 2D video based-motion analysis software. The results show high WIMU^TM^ values for concurrent validity and reliability, when compared with the goniometer and the Kinovea software; examining its use as a reliable and useful instrument for joint mobility measurement. The results state that WIMU^TM^ reported similar angle measurements as the other criterion methods.

Previous studies have used correlation coefficient to assess validity and reliability. However, this coefficient is unable to detect constant error; therefore, this analysis cannot state the validity of an instrument [18]. Bland-Altman plots provide accurate information about the agreement between measures [14]. In our analysis, Bland-Altman plots show that most of the measurements are close to the mean of the differences with both instruments—goniometer and Kinovea—offering a high level of agreement. Additionally, the ICCs and correlation coefficient shows a perfect agreement between measures (1.00).

In this study, we provide both relative and absolute indices of reliability. Both indices showed good reliability for ankle dorsiflexion, when measured using any of the devices (WIMU^TM^, goniometer, or 2D video-analysis with Kinovea), although WIMU^TM^ presented a better absolute reliability index. The SEM was included since its value is very important in the correct clinical interpretation of test results. The SEM is used to indicate the amount of measurement noise, which is unlikely to be of clinical significance. The SEM percentages in the present study suggest that differences of less than 0.64% should be considered measurement noise in ankle dorsiflexion assessed by WIMU^TM^. Whether a post-treatment difference that lies between the SEM and SRD represents a genuine change is less certain [19]. The SRD percentages in the present study suggest that for ankle dorsiflexion assessed by WIMU^TM^, a change of 5.81% is necessary. 

The increase in new instruments and methods to measure angles (joint mobility) for its use in evaluation for rehabilitation or performance in sports has made several studies examine the validity and reliability of these methods. Other studies have stated that new smartphones applications are valid and reliable. The knee goniometer app has shown ICCs’ validity values to be similar to that of WIMU^TM^ (>0.98). The Coach’s Eye is another instrument to assess joint mobility, with ICCs lower than 0.9 [3]; DrG has values of intra and inter-rater reliability >0.86 [20]; the clinometer reported inter-rater reliability of 0.65 [21]. Other methods, such as photography-based goniometry, have shown values of test-retest reliability of 0.93 to 0.99 [22] or Kinovea, with intra and inter-rater reliability ICCs from 0.87 to 0.97 [23]. 

When comparing WIMU^TM^ with previous methods (with goniometer as a criterion), the device was found to have greater reliability, which may be due to several factors. 2D and 3D analysis systems use different markers or reference lines to measure angles. This can cause variations from subject to subject or session to session [24]. In this study, when comparing the results of Phase 1 with Phase 2, it was observed that the latter was lesser, although negligible, valid, and reliable due to the fact that WIMU^TM^ had to be placed on the leg. In addition, 2D devices are dependent on correct plane of the camera for accuracy, which is a drawback [18]. WIMU^TM^ does not require human interaction, only placement of the device on the body, and QÜIKO automatically shows the angle, which is calculated by transformation of unit quaternions into Euler angles. Independent of the instrument used, measurement-related errors can be associated with the tool, the tester, or the ROM [25]. In fact, novice clinicians have reported higher SEMs with the use of goniometers, whereas experienced clinicians have reported higher SEMs with the KGA application [2]. 

Measurements in human movements can be easily automated, considering which, maximum and minimum angles appear automatically in the QÜIKO software. The results point out that WIMU^TM^ have a perfect consistency in angle measurement. Further studies are necessary to assess the validity of this device in real sport movements, postural evaluation, or different clinical conditions.

## 5. Conclusions

The current findings conclude that WIMU^TM^ is a valid and reliable instrument to measure angle positions and could be used for the assessment of joint ROMs. WIMU^TM^, with the help of its software, provides information on angles in real time; it could be used to monitor rehabilitation or training exercises to provide immediate feedback. The WIMU^TM^ does not need cables, so it greatly facilitates the freedom of movement. Although analysis have been done in static positions as dorsiflexion due to research purposes, WIMU^TM^ allows measuring ROM in dynamic movements. WIMU^TM^ can be placed on a specific joint for health testing purposes—in a specific sportive movement in real context. For example, in a jump shot or change of direction in basketball, the test can be conducted with real load of movement, without having to consider placement of the camera or halt movement to fix the goniometer.

## Figures and Tables

**Table 1:** Descriptive statistics and validity analysis based on intraclass correlation coefficients (ICC) and Pearson’s r for validity analysis.

Phase 1	WIMU^TM^	Goniometer	ICC	IC 95%	Pearson’s r
	73.84 ±43.76	74.14 ± 43.64	1.00	1.00–1.00	1.00 *
		Kinovea			
		73.73 ± 44.02	1.00	1.00–1.00	1.00 *
Phase 2	WIMU^TM^	Goniometer	ICC	IC 95%	Pearson’s r
	30.78 ± 2.35	30.68 ± 2.42	0.986 *	0.979–0.991	0.986 *
		Kinovea			
		30.74 ± 2.37	0.997 *	0.996-0.998	0.997 *

* *p* < 0.001; Values in grades.

**Table 2:** Test-retest reliability.

Variables	Mean1 ± SD	Mean2 ± SD	ICC	IC 95%	SEM	%SEM	SRD	%SRD
WIMU^TM^	30.78 ± 2.35	30.62 ± 2.09	0.916 *	0.876–0.944	0.64	2.10	1.78	5.81
Goniometer	30.68 ± 2.42	30.57 ± 2.16	0.901 *	0.853–0.934	0.72	2.36	2.01	6.55
Kinovea	30.74 ± 2.37	30.43 ± 2.20	0.919 *	0.880–0.946	0.65	2.13	1.80	5.89

* *p* < 0.001; SEM: Standard Error of Measurement; values in grades. SRD: Smallest Real Difference. SD: Standard Deviation.
